# Psychosocial experiences of frontline health professionals working in hospitals during the covid-19 pandemic

**DOI:** 10.1192/j.eurpsy.2022.1329

**Published:** 2022-09-01

**Authors:** C. Papathanasiou, M. Tritari

**Affiliations:** Panteion University of Social and Political Sciences, Psychology, Athens, Greece

**Keywords:** Covid-19, healthcare workers, psychosocial experiences, Qualitative research

## Abstract

**Introduction:**

The covid-19 pandemic exerts severe pressure on health systems worldwide and creates stressful working conditions for healthcare workers.

**Objectives:**

The aim of this study, which used the focus group method, was to investigate the psychosocial experiences of the healthcare personnel working in covid-19 wards.

**Methods:**

An interview guide was specially designed and the sample consisted of twelve frontline healthcare workers. Data analysis was based on the empirically grounded theory and thematic analysis was used as a method.

**Results:**

One overarching theme called “Threat” and three main themes were identified: a) Nature of the disease, b) Interpersonal relationships at the hospital, and c) Challenges-Interventions. The concept of “Threat” is dominant throughout the discussion and transcends every issue of the analysis. The first main theme “Nature of the disease” refers to the fear of infection and spread of the virus in the professionals’ environment, the existential concerns brought to the surface by the fear of death, as well as the stigma experienced by health professionals as “potential carriers” of the coronavirus. The second main theme “Interpersonal relationships at the hospital” concerns the relationships developed both among health staff and between health professionals and patients in response to the challenges of the epidemic. The third main theme concerns the obstacles that health professionals face in carrying out their work, the strategies they adopt to deal with stressful situations, but also the type of institutional support they need.

**Conclusions:**

Hospital staff training on the biomedical developments about covid-19 as well as face-to-face self-help groups are recommended.

**Disclosure:**

No significant relationships.

